# Advancing the science on chemical classes

**DOI:** 10.1186/s12940-022-00919-y

**Published:** 2023-01-12

**Authors:** Maricel V. Maffini, Swati D. G. Rayasam, Daniel A. Axelrad, Linda S. Birnbaum, Courtney Cooper, Shari Franjevic, Patrick M. MacRoy, Keeve E. Nachman, Heather B. Patisaul, Kathryn M. Rodgers, Mark S. Rossi, Ted Schettler, Gina M. Solomon, Tracey J. Woodruff

**Affiliations:** 1 Independent Consultant, Frederick, MD USA; 2 Department of Obstetrics, Program on Reproductive Health and the Environment, Gynecology and Reproductive Sciences, University of California, Box 0132, 490 Illinois Street, Floor 10, San Francisco, CA 94143 USA; 3 Scientist Emeritus and Former Director, National Institutes of Environmental Health Sciences and National Toxicology Program, Research Triangle Park, NC USA; 4 Scholar in Residence, Duke University, Durham, NC USA; 5 Clean Production Action, Somerville, MA USA; 6 Defend Our Health, Portland, ME USA; 7 Department of Environmental Health and Engineering, Bloomberg School of Public Health, Johns Hopkins University, Baltimore, MD USA; 8 Johns Hopkins Risk Sciences and Public Policy Institute Bloomberg School of Public Health, Johns Hopkins University, Baltimore, MD USA; 9 Department of Biological Sciences, Center for Human Health and the Environment, North Carolina State University, Raleigh, NC USA; 10 Silent Spring Institute, Newton, MA USA; 11 Science and Environmental Health Network, Ames, Iowa USA; 12 University of California, San Francisco School of Medicine, San Francisco, CA USA; 13 Public Health Institute, Oakland, CA USA

**Keywords:** Chemical class, Regulation, Decision-making, Toxic chemicals, Risk assessment, Chemical grouping, Ortho-phthalates

## Abstract

**Background:**

Hazard identification, risk assessment, regulatory, and policy activity are usually conducted on a chemical-by-chemical basis. Grouping chemicals into categories or classes is an underutilized approach that could make risk assessment and management of chemicals more efficient for regulators.

**Objective and methods:**

While there are some available methods and regulatory frameworks that include the grouping of chemicals (e.g.,same molecular mechanism or similar chemical structure) there has not been a comprehensive evaluation of these different approaches nor a recommended course of action to better consider chemical classes in decision-making. This manuscript: 1) reviews current national and international approaches to grouping; 2) describes how groups could be defined based on the decision context (e.g., hazard/risk assessment, restrictions, prioritization, product development) and scientific considerations (e.g., intrinsic physical-chemical properties); 3) discusses advantages of developing a decision tree approach for grouping; 4) uses ortho-phthalates as a case study to identify and organize frameworks that could be used across agencies; and 5) discusses opportunities to advance the class concept within various regulatory decision-making scenarios.

**Results:**

Structural similarity was the most common grouping approach for risk assessment among regulatory agencies (national and state level) and non-regulatory organizations, albeit with some variations in its definition. Toxicity to the same target organ or to the same biological function was also used in a few cases. The phthalates case study showed that a decision tree approach for grouping should include questions about uses regulated by other agencies to encourage more efficient, coherent, and protective chemical risk management.

**Discussion and conclusion:**

Our evaluation of how classes of chemicals are defined and used identified commonalities and differences based on regulatory frameworks, risk assessments, and business strategies. We also identified that using a class-based approach could result in a more efficient process to reduce exposures to multiple hazardous chemicals and, ultimately, reduce health risks. We concluded that, in the absence of a prescribed method, a decision tree approach could facilitate the selection of chemicals belonging to a pre-defined class (e.g., chemicals with endocrine-disrupting activity; organohalogen flame retardants [OFR]) based on the decision-making context (e.g., regulatory risk management).

**Supplementary Information:**

The online version contains supplementary material available at 10.1186/s12940-022-00919-y.

## Background

In 2009, the National Academy of Sciences (NAS) report Science and Decisions: Advancing Risk Assessment described risk assessment as “a dominant public-policy tool for informing risk managers and the public about the different policy options for protecting public health” [1]. Regulators at federal and state agencies usually conduct risk assessments of chemicals used in commerce on a chemical-by-chemical basis; while this is a well-established method it is also time- and resource-intensive. Because chemical-by-chemical risk assessment has been the standard approach for several decades [2], regulators are prone to adhere to precedent regardless of whether scientific advances render the precedent irrelevant or problematic.

There are several key problems with the current chemical-by-chemical approach to risk assessment. First, there is a tendency to assume that chemicals with insufficient data to estimate either hazard or risk pose no risk, as highlighted in the Science and Decisions report [1]. This assumption allows hazardous chemicals to enter or remain in the marketplace unless they are explicitly prohibited by means other than risk assessment [3]. A second problem is the substitution of hazardous chemicals with others that have similar structure and function (e.g., some bisphenols and brominated flame retardants) but are relatively untested. This often results in a regrettable substitution, a replacement that may be as harmful or more harmful than the original chemical of concern [4]. Finally, single-chemical risk assessment does not capture real-life exposures to mixtures and the potential increased cumulative risk that result from exposures to multiple chemicals (this is further discussed in the companion paper on exposure by Vandenberg et al. in this issue). Consequently, it is likely that the risks associated with multiple chemical exposures are underestimated in the current approach [5].

With tens of thousands of chemicals already in use [6] and ongoing demand for new chemicals and uses, an approach to hazard assessment, risk assessment, and risk management including bans and restrictions, based on groups or classes of compounds is needed. Furthermore, there are many advantages to assessing chemicals as classes including:

- Reducing the tendency to assume that chemicals with no data pose no risk;
- Reducing regrettable substitutions by extrapolating information from data-rich chemicals to data-poor chemicals within the same class;
- Improving risk assessment by considering the cumulative health impacts of exposure to multiple chemicals, thus correcting the underestimation of risk that results from the single-chemical approach;
- Improving public health by reducing exposure to many chemicals of concern at once;
- Increasing efficiency and reducing the use of financial and human resources, resulting in shorter decision-making times;
- Facilitating monitoring of environmental exposures, including biomonitoring;
- Better-informed decision-making throughout the supply chain, including among consumers.

Although the advantages to chemical grouping are many, there are also significant barriers. Unless there is a legal requirement or a clear competitive advantage, agencies and other entities are likely to continue applying familiar and customary approaches. Furthermore, lack of experience in implementing a class approach and lack of established best practices and procedures are challenges to implementation; for example, determining the boundaries of a class can require judgment and could be subject to differing opinions based on choice of criteria and decision context. In the private sector, some companies may have policies for their suppliers indicating certain groups of chemicals are unacceptable in their products, but the complex supply chain, lack of ingredient transparency along the supply chain, and competitive markets are significant barriers to implementation and/or broader adoption of these policies.

The lack of a single definition of class or single method to group chemicals into a class is also a major challenge to greater utilization of the grouping approach. Another problem is that classes created according to one set of criteria (e.g., chemical structure) may be heterogenous in terms of hazard, exposure and use. Heterogeneity within classes can lead to disagreement as to whether the grouping is appropriate. However, the availability of many approaches that can be tailored to specific needs and actions is an advantage that should facilitate adoption of the class approach. Some approaches are very broad and can encompass thousands of chemicals in a class, while others are very narrow and often result in only a handful of chemicals within a class. Regardless of the approach, grouping chemicals into classes for purposes of evaluation and decision-making helps address many of the shortcomings of a single-chemical approach, thus improving public health.

### What are classes of chemicals and how can they be used?

Some classes are already defined in law or regulation. In the United States, the Consumer Product Safety Improvement Act (CPSIA) [7] specifically requires assessment of the health effects of phthalates used in products for children, considering each phthalate individually and in combination with other phthalates. The Food Quality Protection Act requires assessing risk of pesticide residues in foods, considering the cumulative effects of pesticides that have a common mechanism of toxicity [8]. The Clean Air Act identifies hazardous air pollutants that are regulated both on an individual level (such as benzene, di(2-ethylhexyl) phthalate and dimethyl phthalate) and as classes of compounds (such as polycyclic organic matter, glycol ethers, and cadmium compounds) [9]. In other cases, the legal guidance only mentions improving the efficiency of the assessment [10].

In the absence of predetermined classes, regulatory agencies and other organizations have developed methods to group chemicals including:

- Structural similarity (e.g., common chemical group, shared metabolism,precursors, etc.);
- Causes adverse effects on the same organs or biological systems (e.g., nervous system; thyroid gland);
- Causes a similar adverse health outcome regardless of mechanism of action (e.g., cancer, disruption of male sexual development; hypothyroidism);
- Causes toxicity by the same mode of action or molecular mechanism (e.g., inhibition of acetylcholinesterase);
- Similar intrinsic hazard traits (e.g., endocrine disruption);
- Similar physical-chemical characteristics (e.g., persistence, bioaccumulation);
- Common uses or functions (e.g., pesticides, flame retardants); and
- Structurally related chemicals that occur together or are formed by the same process in the environment (e.g., water disinfection byproducts).

Often more than one of these methods is used to jointly define a class. For example, organophosphate pesticides share common functions (i.e., pesticide), structural similarities, and also have similar molecular mechanisms of toxicity (i.e. inhibition of acetylcholinesterase) [11].

Grouping of chemicals in classes or categories has been used in different regulatory contexts such as:

- Cumulative risk assessment: the first step to assess an entire class of chemicals is to identify its members. Based on the criteria for grouping, chemicals could be assigned to a single category or multiple subcategories [4, 12, 13].
- Inference regarding chemical properties: in a defined group, data-poor chemicals are assumed to have similar properties or toxicity as the data-rich members of the same group. This is commonly known as read-across and is used in safety assessments of new chemicals to expedite the process and reduce testing [10, 14]. It is also used in cases of reassessment of prior decisions for a group comprised of a mix of chemicals with and without adequate data [15].
- Prioritization for risk management: assess the relative risk among members of a class in cases of clean-up of contaminated sites, restrict to avoid regrettable substitutions, and establish as low or high priority for risk assessment [16].
- Regulatory disclosure for pollution prevention: listing entire classes in emission inventories (rather than individual constituents of the class) in cases of chemicals released into the water, air, or soil [17].
- Bans: such as in the case of polychlorinated biphenyls (PCBs) [3].

In the last few years, some businesses have responded to health and environmental concerns by replacing entire classes of chemicals. For example, the per- and polyfluorinated alkyl substances (PFAS) class has been voluntarily removed from some articles such as certain popcorn bags, cosmetics, textiles for sportswear and household products [18]. The PFAS class as defined by OECD is based on structural similarity in which there is at least one fully fluorinated carbon [19]. The class of ortho-phthalates (defined by chemical structure as esters of phthalic acid that contain two carbon chains located in the ortho position) has also been targeted for replacement in food packaging and equipment [20]. This is an indication that such an approach is feasible and likely profitable [21].

Given that there are already some identified classes of chemicals, and that grouping can be an effective approach to regulating and reducing chemical exposures, this paper reviews current approaches to identify new groups and address known classes of chemicals. Additionally, this paper identifies best practices for better application of the class approach in a policy and regulatory context.

## Methods

### Information gathering

We evaluated major sources of information including statutes, regulations, and guidance documents to categorize methods used in grouping chemicals into classes. The source selection started with the 2019 NAS report “A Class Approach to Hazard Assessment of Organohalogen Flame Retardants,” which documents several current efforts to assess classes [4]. These efforts were based on statutes, regulatory activities in the US and European Union, and guidance from authoritative bodies including the NAS and the Organization for Economic Co-Operation and Development (OECD). The initial list was then supplemented with examples from additional sources including US state laws and regulations, non-regulatory organizations, and academic publications. The final list (included in Supplementary Materials as Tables S1 and S2) is the result of the authors’ collective knowledge and the NAS report on organohalogen flame retardants and is not meant to be exhaustive. From each source, we extracted information on the organization that developed the grouping method, whether there was a legal requirement for grouping, the scientific consideration on which the group was established, and the method’s implementation, where available.

### Phthalates as a case study for exposure reduction

To illustrate different methods employed by US regulatory agencies, we reviewed the approach to chemical grouping of phthalates by Consumer Product Safety Commision (CPSC) under the Consumer Product Safety Improvement Act (CPSIA), EPA under the Toxic Substances Control Act (TSCA), and FDA under the Food Drug and Cosmetic Act (FDCA). We focused on legal mandates, scientific bases for grouping, and reasons for diverging approaches.

## Results

### There are many available methods for grouping chemicals that can be tailored to specific needs

We identified a total of 19 sources containing information on grouping methods. (Tables S1 and S2) Of these, 13 were available or are under consideration by U.S. domestic—federal and state—and international regulatory agencies (Table 1). In 10 of these instances there was an explicit legal requirement to consider groups or classes of chemicals, but only in three cases did the mandate include consideration of cumulative impacts: EPA and European Food Safety Authority (EFSA) for pesticides and CPSC for phthalates. In two instances, consideration of the category of chemical substances and mixtures are included in the statute and regulations. In the case of the CPSC Federal Hazardous Substances Act, the Commission expressed concern that OFRs as a class present a serious public health issue [32]. Regarding TSCA, section 26C defines a category of chemicals as “a group of chemical substances the members of which are similar in molecular structure, in physical, chemical, or biological properties, in use, or in mode of entrance into the human body or the environment,” and authorizes the EPA to take any action with respect to classes that it can take with respect to individual chemicals or mixtures [23].

**Table 1:** Chemicals grouping methods available to regulatory agencies in the US and the EU

Agency	What was considered	Law	Legal requirement to group chemicals	Rationale for grouping	Grouping applied	Outcome	Reference
US CPSC	Organohalogen flame retardants	Federal Hazardous Substances Act	No	Same function (suppress fire); common chemical structure or predictive biological activity	No	CPSC has yet to agree on NAS grouping proposal	NAS 2019 [4]
US CPSC	Ortho-phthalates	Consumer Product Safety Improvement Act	Yes	Common adverse outcome due to anti-androgenic effects	Yes	Banned eight ortho-phthalates from children’s toys and articles	NAS 2008 [22]
US EPA	Pesticides	Food Quality Protection Act	Yes	Same toxic effect caused by same molecular mechanism of action	Yes	Conducted cumulative risk assessment for five classes of pesticides: organophosphates, N-methyl carbamates, triazines, chloroacetanilides, pyrethrins/pyrethroids	EPA [8]
US EPA	Industrial chemicals	Toxic Substances Control Act	No	Structural similarity; similar “physical, chemical, or biological properties;” similar use; similar exposure pathway	No	EPA has yet to identify classes	EPA [23]
US FDA	Food additives	Food Drug and Cosmetic Act	Yes	Structurally or toxicologically related	Yes	Banned uses of long-chain PFAS in food packaging	FDA [24]
US Washington State	Classes of chemicals in consumer products	Pollution Prevention for Healthy People and Puget Sound Act	Yes	Persistent, bioaccumulative and toxic or hazardous to children’s health	Yes	Regulates five classes of chemicals: PFAS, ortho-phthalates, OFR and other flame retardants identified in RCW 70.240.010, phenolic compounds, PCBs.	Department of Ecology [25]
US Washington State	Per- and polyfluorinated alkyl substances (PFAS)	Packages containing metals	Yes	Persistent, bioaccumulative and toxic	Yes	PFAS banned from use in fiber-based products in contact with food	Department of Ecology [26]
US State of Maine	Chemicals in food packaging and packaging components	Reduction in Toxic Packaging	Yes	Persistent, bioaccumulative and toxic; or hazardous to children’s health	Yes	Bans PFAS and ortho-phthalates	Department of Environmental Protection [27]
US State of California	Chemicals in consumer products	Safer Consumer Products Regulations	No	Persistence	Yes	PFAS	Department of Toxic Substances Control [28]
EU EFSA	Food flavouring substances	Flavouring substances	Yes	Structural similarity	Yes	Regulates flavors across 34 groups	EFSA [29, 9]
EU EFSA	Pesticides	Pesticides	Yes	Common toxic effects	Yes	Cumulative risk assessment; identified two groups: affecting the nervous system and the thyroid system	EFSA [30]
EU ECHA	Industrial chemicals	REACH	Yes	Structural similarity	Yes	Read-across	ECHA [30]
Health Canada	Industrial chemicals		Yes	Structural similarity	Yes	Prioritization of nine groups: aromatic azo- and benzidine-based substances; substituted diphenylamines; cobalt-containing substances; methylenediphenyl diisocyanates and diamines (MDI/MDA); certain internationally classified substances with potential for exposure to individuals in Canada; selenium-containing substances; certain organic flame retardants; ortho-phthalates; and boron-containing substances.	Health Canada [31]

Among the regulatory agencies, structural similarity was the most common grouping method albeit with some variations in definition. In general, the grouping methods include:

1. a common functional group (i.e., chemical similarity within the class); or
2. common precursors and/or likelihood of common breakdown products through physical and/or biological processes which result in structurally similar degradation products (i.e., similarity through biotransformation); or
3. a constant pattern of the properties across the group (e.g., of physicochemical and/or biological properties) or
4. a common mode or mechanism of action or adverse outcome pathway; or
5. common constituents (e.g., similar carbon-chain length).

Among the frameworks, there is a common assumption that structurally similar chemicals have similar toxic effects, therefore a read-across framework can be applied. For instance, the European Chemicals Agency (ECHA) included the read-across framework in its standard testing regime [30]. Structurally similar chemicals are grouped and information requirements for physicochemical, human health, and/or environmental properties can be predicted from tests conducted on reference substance(s) within the group. This approach aims at increasing regulatory efficiency and decreasing use of time and resources. Although read-across is efficient, it relies on the assumption that the untested chemicals within the class are not likely to be significantly more toxic than the ‘anchor’ chemical.

**Fig. 1:**
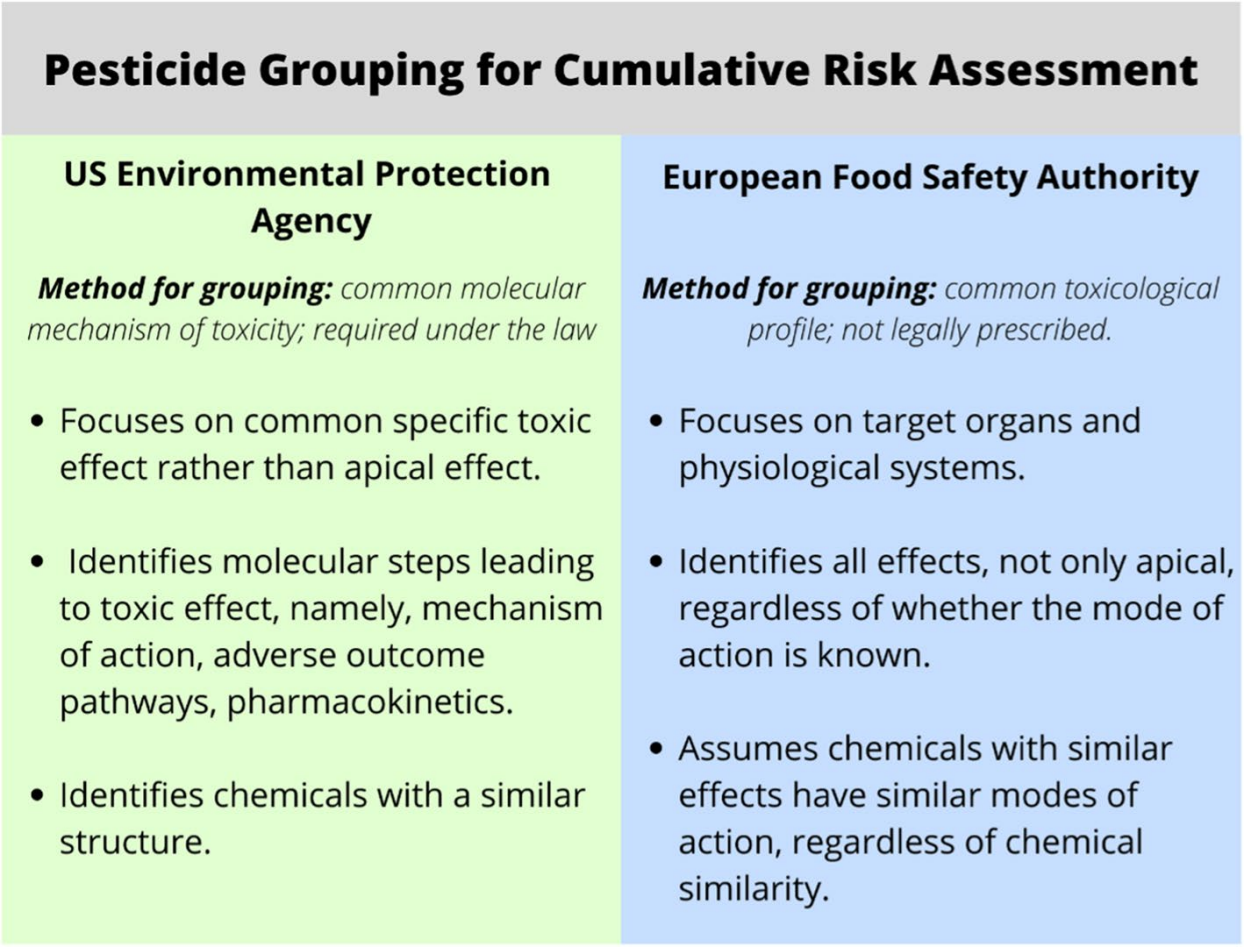
US EPA’s and EU EFSA’s regulatory approaches for grouping pesticides in food for cumulative risk assessment

The second most common chemical grouping identified was based on hazard properties. These range from very broad inclusion criteria such as EFSA’s grouping of pesticides to the narrowly defined groupings of pesticide residues in or on food by the EPA. Figure 1 summarizes the differences between the agencies’ grouping criteria, the main being that the EPA is required to follow a method established by law, while EFSA chooses a grouping mechanism that more adequately meets its regulatory goals [8, 33].

Between these methodological bookends, there are methods based on chemicals that share a hazard property (e.g., anti-androgenicity) and common adverse effects (e.g., altered male reproductive development), but may not share the same molecular mechanism of action [22]. Among the non-regulatory organizations, structural similarity was also the most common grouping approach either used or recommended as shown in Table 2.

**Table 2:** Grouping methods developed or used by non-regulatory organizations

Organization	Chemical/class	Grouping method
Organization for Economic Co-operation and Development	Chemicals in general [34]	Structural similarity
National Academy of Sciences	Phthalates [22]	Common adverse outcomes—anti-androgenicity regardless of mechanism of action
California Environmental Contaminant Biomonitoring Program	Twelve chemical groups have been added to the biomonitoring program [35]	Structural and functional characteristics
US Cosmetic Ingredient Review	Cosmetic products [36]	Structural similarity and physical-chemical properties
Flavor and Extract Manufacturer Association	Flavoring substances, spices [37]	Structural similarity and metabolic fate
Joint Expert Committee on Food Additives (WHO/FAO)	Food ingredients, chemicals and contaminants [38]	Structural similarity

Emerging models to compile and synthesize the growing body of data from new in vitro testing technologies have the potential to be useful [39]. Examples of these include adverse outcome pathways (AOPs) and key characteristics (KCs), which compile and organize mechanistic data used for different purposes. For instance, AOPs seek to identify molecular steps, or “key events” required to produce a toxic effect after exposure to a chemical [40–42]. It has been suggested that AOPs could be used to establish chemical categories that share a toxicity mechanism or common key events [43]. In a recent publication, Andreas Kortenkamp applied the concept of AOP networks (groupings of intersecting AOPs) to identify a diverse set of chemicals, in addition to phthalates, that are predicted to contribute to disorders in male reproductive development [12]. This approach overcomes a narrow focus on structure and molecular mechanism of action. AOP networks can be used to derive criteria for groups of chemicals contributing to the same health outcome but exhibiting diverse chemical structures and modes of action.

With an initial focus on cancer, the concept of KCs was developed as a basis for organizing mechanistic data from diverse chemicals associated with the same health outcome. KCs combine phenotypic data from human and animal studies with mechanistic data. Examples of KCs are receptor ligand or agonist, epigenetic alterations, hormone synthesis, alter immune function, alter cell-cell interactions, alter DNA repair or cause genomic instability, induce chronic inflammation, alter cell proliferation, death, or nutrient supply. Thus far, KCs for carcinogens, endocrine disruptors, and female and male reproductive toxicants have been identified [44–47].

## Discussion

### Ortho-phthalate case study: a missed opportunity to protect the public

Although ortho-phthalates are quickly eliminated from the body, most Americans tested have ortho-phthalates metabolites in their urine daily due to their widespread presence in food, cosmetics, household products, and other sources. In 2008, the US Congress passed the CPSIA giving CPSC authority to permanently ban three ortho-phthalates—di(2-ethylhexyl) phthalate (DEHP), butylbenzyl phthalate (BBP), and dibutylbenzyl phthalate (DBP)— and placed an interim restriction on diisononyl phthalate (DINP), diisodecyl phthalate (DIDP), and di-n-octyl phthalate (DNOP) from children’s toys and childcare articles. These actions became effective in 2008.

In their analysis of 2001–2010 NHANES biomonitoring data; which includes two years after the CPSC ban of the three ortho-phthalates, Zota and colleagues showed the positive and negative outcomes of the enacted public policy [48]. On the positive side, population exposure, as indicated by measurement of urinary ortho-phthalate metabolites in a representative sample of the general population, had decreased. This was an expected outcome considering that in the years before the CPSC restriction in toys and childcare articles, there had been public pressure campaigns and additional restrictions placed in the European Union, which collectively may have resulted in the reduced exposure observed in the US population [49, 50]. The negative outcome was that authors observed an increase in exposure to ortho-phthalates structurally similar to those facing regulatory pressure, e.g., increases in DIBP as DBP declined, revealing a challenge in the implementation of the chemical classes, namely, how expansive a class should be and how to identify chemicals that belong to a class.

CPSC’s Chronic Hazard Advisory Panel (CHAP) also conducted a review of the health effects of ortho-phthalates using a grouping approach based on anti-androgenic activity and cumulative risk assessment [51]. Based on panel’s analysis, CPSC identified a total of eight ortho-phthalates that are now restricted from use in children’s toys and childcare articles to protect the health of children (Table 3).

**Table 3:** Divergent approaches of three federal agencies to the same class of chemicals

Ortho-phthalate	CPSC (children’s toys and childcare articles)	CPSC (all other consumer products)	FDA	EPA
**DPENP**	Banned	Allowed	N/A	N/A
**DEHP**	Banned	Allowed	Allowed	Under Consideration
**BBP**	Banned	Allowed	Allowed	Under Consideration
**DBP**	Banned	Allowed	Allowed	Under Consideration
**DHEXP**	Banned	Allowed	N/A	N/A
**DCHP**	Banned	Allowed	Allowed	Under Consideration
**DINP**	Banned	Allowed	Allowed	Under Consideration
**DIBP**	Banned	Allowed	Allowed	Under Consideration

*N/A *not applicable. No record has been found that FDA has authorized the uses of DPENP and DHEXP in food; EPA has not selected these chemicals for priority risk evaluation. EPA has also selected diisodecyl phthalate (DIDP), which is allowed under FDA, and phthalic anhydride for priority risk evaluation. FDA regulates oral exposure to ortho-phthalates used in food contact articles. EPA regulates industrial uses of ortho-pthalates with various routes of exposures

Unfortunately, the actions by CPSC did not lead to FDA, EPA or even CPSC for uses other than toys and children articles to consider a reevaluation of the safety of these ortho-phthalates, in part due to the lack of statutory requirements. This resulted in insufficient protection of children’s health from ortho-phthalates exposures.

FDA has yet to reevaluate the safety of the ortho-phthalates CPSC acted on [52]. The agency has continued to allow six of the CPSC banned substances (out of a total of 28 ortho-phthalates approved by the agency) to be used in articles in contact with food such as packaging and food processing equipment (e.g., tubing, conveyor belts, sealing gaskets). The FDA does not monitor food for ortho-phthalate content to estimate exposure and trusts manufacturers to self-police by adhering to good manufacturing practices based on product performance (i.e., only use the amount of phthalate needed and no more) rather than on health protection. Although the FDA is required by law [24] to assess the cumulative effects of chemically- or toxicologically related substances in the diet, the agency has only assessed phthalates one at a time. Table 3 outlines the divergent approaches of these three federal agencies (CPSC, FDA, EPA) to the same class of chemicals, showing how chemicals banned in children’s toys and childcare articles continue to be allowed in foods and other consumer products without limitations to exposure.

In 2009 (and revised in 2012), the EPA issued a plan to coordinate with CPSC and the FDA to take action to address “the manufacturing, processing, distribution in commerce, and/or use” of eight ortho-phthalates under its TSCA authority [53]. The agency was concerned about potential high exposure to individual or multiple ortho-phthalates, considering their high production volume and hazard properties. No further action appears to have been taken until 2019 after TSCA was amended to require the EPA to evaluate risks for existing chemicals. At this point in time, the EPA designated five ortho-phthalates as high-priority substances for risk evaluation and two additional ortho-phthalate risk evaluations were initiated based on manufacturer requests [54]. Thus far, it appears that EPA is conducting single-chemical risk evaluations [55].

A lack of regulatory and legal requirements to consider classes means that regulatory agencies do not have to adopt class-based methods and are often paying attention to other priorities. Even within the same agency, different divisions may act independently and without coordination. For example, FDA regulates ortho-phthalates uses in food, in drugs and in medical devices and there seems to be little attempt to coordinate across the FDA centers with responsibility in each area. Additionally, it is scientifically appropriate that chemicals considered hazardous in children’s toys should be anticipated to be hazardous in other products, and on this basis agencies should reevaluate their risk to better protect the population.

### A decision tree concept to advance the science of chemical classes

There is not a single approach to establish a class (Table 1). We propose designing an approach by which any chemical could be assigned to a class or category using a decision tree framework grounded in two equally important components, namely the decision context and scientific considerations. The goal of the decision tree is to help develop the best possible class to achieve a health-protective regulatory outcome that avoids regrettable substitutes. While we focus on its use in a regulatory context, the decision tree could also be applied in the private sector.

The decision-making process should be transparent as practitioners are expected to evaluate what method would be the most appropriate to achieve the regulatory outcome, justify the choice of method(s), and publish the decision. The decisionmaker- could be a business, institution, or government agency and the decision could be policy- or practice-oriented (Table 4). For example, a business could be making manufacturing or purchasing decisions based on chemical classes. A school, university, or hospital could make purchasing decisions based on chemical classes. A government agency could use a class-based approach to develop a prioritization system for assessing existing chemicals. The scope of authority and willingness to consider more visionary class-based approaches will influence the definition of class boundaries for specific purposes. Health-protective new laws would include strong legal frameworks and be flexible enough to adapt to scientific advances.

**Table 4:** Decision context – policy & practice

*Scope of Authority*	
*Visionary*	*Constrained*
• Progressive business model • Chemical prioritization for assessment, replacement, or elimination • Health-protective new laws with flexibility to adapt to scientific advances • Updated regulations	• Laws and regulations which require or allow classes • Laws and regulations which don’t exclude classes • Rigid statutory and regulatory language with specific definitions of what may be included in a class

**Fig. 2:**
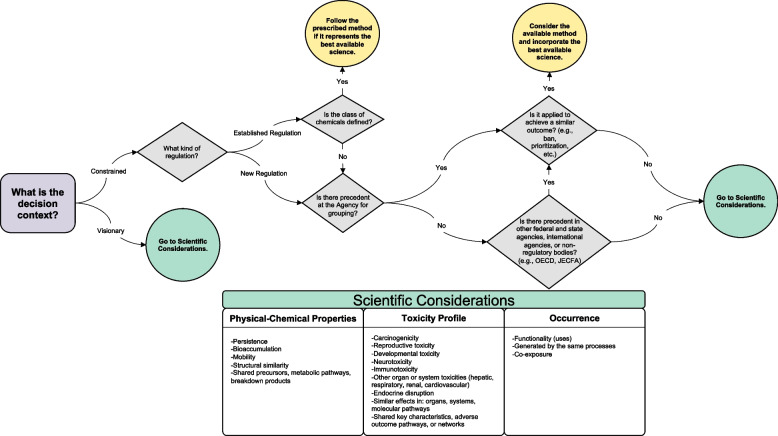
Generic decision tree for chemical grouping

The generic decision tree depicted in Fig. 2 was developed to help guide the process of identifying whether grouping chemicals in a class is possible, if there is precedent for grouping at the agency or sister agencies that could be informative, and which science-based method(s) would work best to achieve a regulatory outcome(s). Regarding scientific considerations, precedent at the agency itself or other regulatory sister agencies’ successful use of grouping methods will help to shape the chemical group. In the private sector, previously successful business models as well as regulatory and consumer pressure will inform decisions regarding scientific basis for the class of chemicals to address. In Table 5, we identified three major categories that could inform grouping and provide some examples for each of them.

**Table 5:** Scientific considerations

*Physical-chemical properties*	*Toxicity Profile*	*Occurrence*
• Persistent • Bioaccumulative • Mobile • Structurally similar • Shared precursors, metabolic pathways, breakdown products	• Carcinogenicity • Reproductive toxicity • Developmental toxicity • Neurotoxicity • Immunotoxicity • Other organ or system toxicities (hepatic, respiratory, renal, cardiovascular) • Endocrine disruption • Similar effects in: organs, systems, molecular pathways • Shared key characteristics, adverse outcome pathways or networks	• Functionality (uses) • Generated by same processes • Co-exposure

The list of scientific considerations was compiled from the methods identified in Tables 1, S1, and S2 and supplemented with relevant scientific knowledge to better protect human and environmental health. Some up-to-date examples include mobility of chemicals in the environment, co-exposures (e.g., mixtures), and mechanistic data-driven concepts such as KCs and AOPs. The proposed decision tree contains a series of yes/no questions developed within a particular context by combining available data and a specific purpose for which the chemical class is being developed. All available data should be considered including physical-chemical properties, toxicity, pharmacokinetic/pharmacodynamic, environmental fate, biomonitoring, epidemiological and clinical studies, etc.

For example, the EPA is evaluating eight phthalates under TSCA [56]. These are di-isobutyl phthalate (DIBP), dicyclohexyl phthalate (DCHP), dibutyl phthalate (DBP), butyl benzyl phthalate (BBP), diethylhexyl phthalate (DEHP), di-isononyl phthalate (DINP), di-isodecyl phthalate (DIDP) and phthalic anhydride which is a precursor. We can then apply the proposed decision tree as in Fig. 1:

1. What is the decision context? *Constrained under TSCA*
2. Are there established regulations? *Yes*
3. Is the class defined? *No*
4. Is there a precedent at the agency for grouping these chemicals? *No*
5. Is there a precedent in other agencies? *Yes, at sister agency CPSC*
6. Is the grouping method applied to meet a similar regulatory need? *Yes, cumulative risk assessment*

Therefore, the EPA could apply a similar grouping method, keeping in mind that, under TSCA, Congress expressly recognized a broad set of effects that may present unreasonable risk of injury to health or the environment, including “carcinogenesis, mutagenesis, teratogenesis, behavioral disorders, [and] cumulative or synergistic effects.” [3] EPA could also consider whether there are additional ortho-phthalates that are also members of the same class.

If we consider a scenario where there is no precedent for regulatory outcome using a class approach, the ortho-phthalates in Table 3 could be grouped based on various scientific considerations which can be used individually or in combination. For example,

1. Structural similarities: DIBP, DCHP, DBP, BBP, DEHP, DINP and DIDP share
  - Chemical groups: esters of phthalic acid
  - Precursor: phthalic anhydride
  - Metabolic pathways
2. Shared hazard properties: DIBP, DCHP, DBP, BBP, DEHP, DINP are toxic to male reproductive development and are endocrine disruptors (DIDP has not been included because “evidence of endocrine disruption in experimental animal studies has not been found” (according to the CHAP convened by CPSC [57].)
3. Are found in same monitoring samples: DIBP, DCHP, DBP, BBP, DEHP, DINP and DIDP share structurally similar urinary metabolites due to shared metabolic pathways

As discussed earlier, in the absence of a prescribed method, structural similarity is the most frequently used method because chemical structures are almost always available, and, in some cases, are the only data available. If only a few members of a class of structurally similar chemicals have hazard information, the general assumption has been that the hazard data for the few applies to other members of the class [15, 30].

### A path forward

Science has advanced on how we measure chemical exposures, test for toxicity, and model and measure health outcomes, but methods for conducting risk assessment and developing the evidence used in chemical policy decision-making has not kept pace. There is well-supported evidence showing health risks from common everyday cumulative exposures to harmful chemicals. Some actions have been taken to address these health risks, either by a Congressionally-directed ban of an entire class such as polychlorinated biphenyls, by use of agencies’ discretionary legal authorities, or in response to consumer pressure [3, 7–9, 15, 58]. In some instances, the efficiency of assessments has increased leading to potential improvement in public health protection. One major outcome of advancing the science of chemical classes is that the targeted allowable exposure to the members of the class would be reduced, namely, instead of several individual reference doses or tolerable daily intakes (TDI), there would be one for the group. For example, the 2019 EFSA reevaluation [59] of DBP, BBP, DEHP, DINP and DIBP established a single TDI for a group of four ortho-phthalates (DBP, BBP, DEHP, DINP) based on similar adverse outcome. The highest individual TDI of the members of the group was 150 μg/kg body weight/day. The new group-TDI is 50 μg/kg body weight/day, a significant reduction in exposure to anti-androgenic ortho-phthalates. Unfortunately, little has been done to develop broad policies and practices to better reflect how groups of chemicals affect health with the goal of reducing harmful exposures, improving public health, and addressing equity in a more resource efficient manner. Furthermore, most agencies continue to conduct risk assessments one substance at a time.

The ortho-phthalates case study revealed some of the challenges to advance the science of chemical classes. In general, agencies strongly adhere to precedent, a feature that when combined with external pressure to preserve the status quo, only reinforces the challenge to change. For instance, in 2019, the EPA initiated TSCA risk evaluations of seven ortho-phthalates. Instead of treating the chemicals as a class or category, as allowed under the law, the EPA has thus far indicated it plans to evaluate them one-by-one. A class approach could increase the efficiency of the evaluation process and better protect public health. Importantly, in 2008 the NAS recommended (in a report the EPA itself had commissioned) that the EPA conduct a cumulative risk assessment of ortho-phthalates as a group [22]. The EPA has not provided any rationale for not implementing this recommendation [55]. Another challenge is the inconsistent approach across agencies to managing the same group of chemicals. This is a lost opportunity to reduce exposures and improve public health and health equity [7]. Lastly, agencies should have flexibility in establishing a class of chemicals to account for scientific advances. When the CHAP on ortho-phthalates was established by CPSC, it defined the class ortho-phthalates based on whether there was evidence of anti-androgenic properties, a decision made by the expert panel, not directed by the law. After the CHAP completed its evaluation, eight ortho-phthalates were included in the group that was banned by CPSC for use in children’s toys and childcare articles. However, in its 2014 report, the CHAP acknowledged that there were other health outcomes (e.g., neurodevelopmental toxicity) shared by some ortho-phthalates [51]. Since then, the evidence of neurodevelopmental toxicity has only grown stronger [58]. Flexibility should be an integral constituent in the development of class approaches to ensure the outcome is based on up-to-date scientific evidence and is the most health protective.

We believe that the concept of a decision tree provides the elements to advance the science of chemical class approaches:

- Any chemical could be assigned to a class or category when applying the decision tree concept. This eliminates the perceived barrier of lack of a single universal method as documented in Table 1 with multiple variations in approaches to establish and manage chemical classes.
- There is flexibility to select already available and tested methods or develop a brand-new approach to implementing existing laws, scientific advances, renewing public health protections, and ensuring equitable outcomes. Grounding the decision tree in both the context in which a decision is made and scientific considerations that evolve with advances in science provides adaptability.
- An agreed-upon decision approach provides an opportunity to stimulate coordination and collaboration across agencies regulating the same chemicals because it incorporates questions about existing grouping methods within and outside an agency.

## Conclusion

There are multiple available methods to use a class approach that meets users’ needs including assessing chemical safety, disclosure of chemical releases, selection of biomonitoring chemicals, and manufacturing and product stewardship. The statutes and regulations we examined allow grouping chemicals for risk assessment and, in some cases, grouping is a legal requirement. More importantly, we have not found explicit exclusion of using a class approach or a legal requirement to conduct chemical-by-chemical risk assessments. There are ongoing successful cases of the class approach, although it continues to be underutilized. The case study indicates that it is implementable as an approach to address harmful exposures more efficiently.

Finally, we argue that the option of grouping chemicals in classes or categories should be explored and used whenever possible for hazard identification, risk assessment, and regulatory or policy activity. Evaluating chemicals in this way will more efficiently and effectively gather data and identify chemicals of concern that pose unacceptable risks. We propose using a flexible decision tree approach based on the decision context and scientific considerations as an opportunity to systematically incorporate chemical classes into the decision-making process.

## Supplementary Information

**Additional file 1: Table S1.** Laws and regulations consulted to inform this study. **Table S2.** Non-regulatory organizations consulted to inform this study.

## Data Availability

Data sharing is not applicable to this article as no datasets were generated or analyzed during the current study.

## Abbreviations

OFR: Organohalogen Flame Retardant
NAS: National Academy of Sciences
CPSIA: Consumer Product Safety Improvement Act
PCBs: Polychlorinated Biphenyls
PFAS: Per- and Polyfluorinated Alkyl Substances
OECD: Organization for Economic Co-Operation and Development
CPSC: Consumer Product Safety Commision
FDCA: Food Drug and Cosmetic Act
TSCA: Toxic Substances Control Act
EFSA: European Food Safety Authority
ECHA: European Chemicals Agency
AOPs: Adverse Outcome Pathways
KCs: Key Characteristics
DEHP: Di(2-ethylhexyl) phthalate
BBP: Butylbenzyl phthalate
DBP: Dibutylbenzyl phthalate
DINP: Diisononyl phthalate
DIDP: Diisodecyl phthalate
DNOP: Di-n-octyl phthalate
CHAP: Chronic Hazard Advisory Panel
FDA: Food and Drug Administration
DPENP: Di-n-pentyl phthalate
DHEXP: Di-n-hexyl phthalate
DIBP: Di-isobutyl phthalate
DCHP: Dicyclohexyl phthalate
DEHP: Diethylhexyl phthalate
TDI: Tolerable Daily Intakes
