# Supplementary material for: Advancing the science on chemical classes
Source: Environ Health. 2023 Jan 12;21(Suppl 1):120. doi: 10.1186/s12940-022-00919-y (PMC9835214; doi:10.1186/s12940-022-00919-y)
Supplement: Supplementary file 1 — Additional file 1: Table S1. Laws and regulations consulted to inform this study. Table S2. Non-regulatory organizations consulted to inform this study. [file 12940_2022_919_MOESM1_ESM.docx]

**Supplementary Information**

**Table S1:** Laws and regulations consulted to inform this study

| **Act** | **Implementing Agency** | **Year** |
| --- | --- | --- |
| *US Federal Laws and Regulations* | | |
| Federal Hazardous Substances Act [1] | Consumer Protection Safety Commission | 1960 |
| Consumer Product Safety Improvement Act [2] | Consumer Protection Safety Commission | 2008 |
| Food Quality Protection Act [3] | Environmental Protection Agency | 1996 |
| Frank Lautenberg Chemical Safety for the 21st Century Act [4] | Environmental Protection Agency | 2016 |
| Food Additive Amendment to the Federal Food Drug and Cosmetic Act [5] | Food and Drug Administration | 1958 |
| *US State Laws and Regulations* | | |
| Washington State Pollution Prevention for Healthy People and Puget Sound Act [6] | Department of Ecology | 2019 |
| Washington State Packages Containing Metals [7] | Department of Ecology | 2018 |
| State of Maine Reduction in Toxic Packaging Law [8] | Department of Environmental Protection | 2019 |
| California Safer Consumer Products Regulations [9] | Department of Toxic Substances | 2013 |
| *International Laws and Regulations* | | |
| Regulation (EC) No. 396/2005 of the European Parliament and the Council on maximum residue levels of pesticides in or on food and feed of plant and animal origin and  amending Council Directive 91/414/EEC [10] | European Food Safety Authority | 2005 |
| Regulation (EC) No. 1907/2006 of the European Parliament and the Council concerning the Registration, Evaluation, Authorisation and  Restriction of Chemicals (REACH), establishing a European Chemicals Agency, amending Directive 1999/45/EC and repealing Council Regulation (EEC) No 793/93 and Commission Regulation (EC) No 1488/94 as well as  Council Directive 76/769/EEC and Commission Directives 91/155/EEC, 93/67/EEC, 93/105/EC and 2000/21/EC [11] | European Chemical Agency | 2006 |
| Regulation (EC) No 1334/2008 and EC 1565/2000 of the European Parliament and of the Council on flavourings and certain food ingredients with flavouring properties for use in and on foods [12] [13] | European Food Safety Authority | 2008, 2000 |
| Canadian Environmental Protection Act [14] | Environment and Natural Resources | 1999 |

**Table S2:** Non-regulatory organizations consulted to inform this study

| **Organization** | **Activity** |
| --- | --- |
| Organization for Economic Co-Operation and Development | Published the Guidance on Grouping of Chemicals. [15] |
| World Health Organization/Food and Agriculture Organization (WHO/FAO) Joint Expert Committee on Food Additives (JECFA) | Performs risk assessments and provides advice to FAO, WHO and their member countries on chemicals safety. [16] |
| US National Academy of Sciences | Published the report Phthalates and Cumulative Risk Assessment: The task ahead [17]  Published the report A Class Approach to Hazard Assessment of Organohalogen Flame Retardants. [18] |
| U.S. Cosmetic Ingredient Review (CIR) | Conducts safety reviews of ingredients used in cosmetics in the U.S. [19] |
| Flavor and Extract Manufacturers Association (FEMA) | Performs safety assessment of flavouring substances. [20] |
| California Department of Public Health | California Environmental Contaminant Biomonitoring Program designate chemicals that are known to, or strongly suspected of, adversely impacting human health or development for biomonitoring. [21] |

**References**

1. **Federal Hazardous Substances Act**. In: *15 USC.* USA; 1960.

2. **Consumer Product Safety Improvement Act of 2008**. In*.* USA; 2008.

3. **Food Quality Protection Act**. In: *7 USC.* vol. 7. USA; 1996.

4. **Frank R. Lautenberg Chemical Safety for the 21st Century Act**. In*.*; 2016.

5. **Food Additive Amendment to the Federal Food, Drug, and Cosmetic Act**. In*.*, vol. 21 U.S.C. USA; 1958.

6. **Pollution Prevention for Healthy People and Puget Sound Act**. In: *RCW.* vol. Title 70A; 2019.

7. **Packages Containing Metals**. In: *RCW.* vol. Title 70; 2018.

8. **Reduction in Toxic Packaging**. In: *MRS* vol. Title 32 2019.

9. **Safer Consumer Products**. In: *CCR.* vol. CCR Title 22, Division 4.5; 2013.

10. **Maximum residue levels of pesticides in or on food and feed of plant and animal origin**. In*.*, vol. EC 396/2005; 2005.

11. **Registration, Evaluation, Authorisation and Restriction of Chemicals (REACH)**. In*.*, vol. EC 1907/2006; 2006.

12. **Flavourings and certain food ingredients with flavouring properties for use in and on foods**. In*.*, vol. EC 1334/2008; 2008.

13. **Evaluation programme for flavouring substances**. In*.*, vol. EC 1565/2000; 2000.

14. **Canadian Environmental Protection Act**. In: *SC.* vol. c. 33. Canada; 1999.

15. Co-operation OfE, Development: **Guidance on grouping of chemicals**: Organisation for Economic Co-operation and Development; 2014.

16. **Joint FAO/WHO Expert Committee on Food Additives (JECFA)** [<https://www.who.int/foodsafety/areas_work/chemical-risks/jecfa/en/>]

17. Council NR: **Phthalates and cumulative risk assessment: the tasks ahead**. 2009.

18. National Academies of Science E, and Medicine: **A Class Approach to Hazard Assessment of Organohalogen Flame Retardants**. In*.* Washington (DC); 2019.

19. **Cosmetic Ingredient Review**

20. **Flavor and Extract Manufacturers Association** [<https://www.femaflavor.org/>]

21. Program CECB: **Biomonitoring California**. In*.* Edited by Health TCDoP, vol. Division 103, Part 5, Chapter 8. California Health and Safety Code.
